# Reduction of Cross-Reactive Carbohydrate Determinants in Plant Foodstuff: Elucidation of Clinical Relevance and Implications for Allergy Diagnosis

**DOI:** 10.1371/journal.pone.0017800

**Published:** 2011-03-14

**Authors:** Heidi Kaulfürst-Soboll, Melanie Mertens, Randolf Brehler, Antje von Schaewen

**Affiliations:** 1 Institute of Plant Biology and Biotechnology, University of Münster, Münster, Germany; 2 Department of Dermatology, University of Münster, Münster, Germany; Université Paris Descartes, France

## Abstract

**Background:**

A longstanding debate in allergy is whether or not specific immunoglobulin-E antibodies (sIgE), recognizing cross-reactive carbohydrate determinants (CCD), are able to elicit clinical symptoms. In pollen and food allergy, ≥20% of patients display *in-vitro* CCD reactivity based on presence of α1,3-fucose and/or β1,2-xylose residues on *N-*glycans of plant (xylose/fucose) and insect (fucose) glycoproteins. Because the allergenicity of tomato glycoallergen Lyc e 2 was ascribed to *N*-glycan chains alone, this study aimed at evaluating clinical relevance of CCD-reduced foodstuff in patients with carbohydrate-specific IgE (CCD-sIgE).

**Methodology/Principal Findings:**

Tomato and/or potato plants with stable reduction of Lyc e 2 (tomato) or CCD formation in general were obtained via RNA interference, and gene-silencing was confirmed by immunoblot analyses. Two different CCD-positive patient groups were compared: one with tomato and/or potato food allergy and another with hymenoptera-venom allergy (the latter to distinguish between CCD- and peptide-specific reactions in the food-allergic group). Non-allergic and CCD-negative food-allergic patients served as controls for immunoblot, basophil activation, and ImmunoCAP analyses. Basophil activation tests (BAT) revealed that Lyc e 2 is no key player among other tomato (glyco)allergens. CCD-positive patients showed decreased (re)activity with CCD-reduced foodstuff, most obvious in the hymenoptera venom-allergic but less in the food-allergic group, suggesting that *in-vivo* reactivity is primarily based on peptide- and not CCD-sIgE. Peptide epitopes remained unaffected in CCD-reduced plants, because CCD-negative patient sera showed reactivity similar to wild-type. In-house-made ImmunoCAPs, applied to investigate feasibility in routine diagnosis, confirmed BAT results at the sIgE level.

**Conclusions/Significance:**

CCD-positive hymenoptera venom-allergic patients (control group) showed basophil activation despite no allergic symptoms towards tomato and potato. Therefore, this proof-of-principle study demonstrates feasibility of CCD-reduced foodstuff to minimize ‘false-positive results’ in routine serum tests. Despite confirming low clinical relevance of CCD antibodies, we identified one patient with ambiguous *in-vitro* results, indicating need for further component-resolved diagnosis.

## Introduction

Specific immunoglobulin-E antibodies (sIgE) directed against plant-derived carbohydrate epitopes (cross-reactive carbohydrate determinants, CCD [1]) are ubiquitous among patients with confirmed pollen or food allergy (reviewed by Altmann) [2]. At least 20% of patients with tomato, carrot or celery allergy exhibit CCD-sIgE in their sera [3]–[6]. The main motifs of these carbohydrate epitopes are asparagine (*N*)-linked glycan chains carrying *core* α1,3-fucose and β1,2-xylose residues [7]. They form essential parts of two independent complex *N*-glycan epitopes found on glycoproteins of plants and lower animals, and occur in pollen, natural rubber latex, vegetables and fruits, hymenoptera venoms (only α1,3-fucose), and in some pathogenic worms but not in mammals (see Altmann) [2]. Therefore, patients with CCD-sIgE display a broad range of cross-reactions when subjected to serum investigations. In non-allergic persons, CCD-sIgE levels are usually below detection limits [8], [9].

During the past decades, several investigations have been conducted on carbohydrate-sIgE antibodies concerning their ability to elicit allergic symptoms. Because sufficient evidence for their clinical relevance in pollen, food or hymenoptera-venom allergy is still lacking, CCD epitopes are mainly regarded to obscure *in-vitro* detection of true allergens [2], [8], [10]–[13]. On the other hand, some authors have concluded from their studies that in pollen- or plant food-allergic patients (e.g. with symptoms to cypress pollen, tomato, or celery) carbohydrate-specific IgE antibodies may be responsible for the allergic reactions: first, because basophil activation - a crucial type I-allergic event - was observed with native, glycosylated but not with recombinant, non-glycosylated allergens expressed in *Escherichia coli* (*E. coli*), and second, because patient sera predominantly contained CCD-sIgE [7], [14]–[16].

In potato and tomato food allergy, several glycosylated allergens are known [16]–[20]. Among them are in potato (*Solanum tuberosum*), Sola t 1 (patatin, the main storage protein of tubers) decorated with up to three heterogeneous complex *N*-glycans [21]–[25], and Sola t 2 (a glycoprotein belonging to the family of soybean trypsin inhibitors [17]). Interestingly in tomato (*Lycopersicon esculentum* alias *Solanum lycopersicum*), only Lyc e 2 (vacuolar β-fructofuranosidase or invertase, a sucrose splitting enzyme appearing during fruit ripening [16], [18], [19], [26], [27]), is listed as glycoallergen in the official World Health Organization/ International Union of Immunological Societies (WHO/IUIS) allergen data base, although more glycoproteins of tomato are known to bind specific IgE (e.g. polygalacturonase 2A [18], [19], peroxidase I [28], and pectin(methyl)esterase [18], [19]). Importantly, allergenic reactivity of Lyc e 2 (with four potential *N*-glycosylation sites) seems to depend on CCD epitopes alone [7], [16].

**Figure 1:**
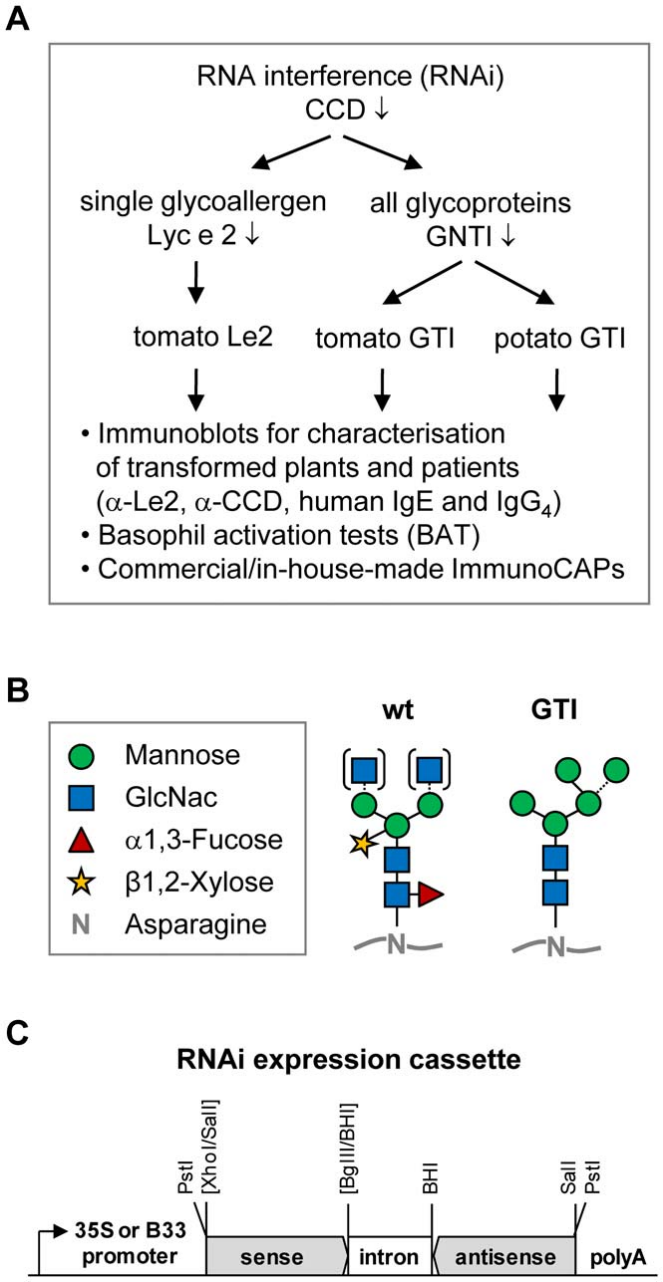
Schematic of this studies' approach. **A**: Flow chart of conducted experimental analyses. The impact of single glycoallergen Lyc e 2 in tomato allergy was studied by establishing Lyc e 2-reduced tomato fruits (plants further referred to as Le2). To evaluate contribution of carbohydrate- *versus* peptide-specific determinants in reactivity to foodstuff, CCD-reduced tomato and potato plants were created by silencing N-acetylglucosaminyltransferase-I (GNTI, plants further also referred to as GTI), catalyzing the crucial step leading to CCD formation in the Golgi apparatus. **B**: Predominant protein-bound *N*-glycan structures prevailing in wild-type (wt) and in GTI transformants (no CCD epitopes). *N*-glycan structures are depicted according to the proglycan system (www.proglycan.com). Note that terminal GlcNac (N-acetylglucosamine) residues are not present on fully trimmed wild-type *N*-glycans (dotted lines and brackets). **C**: RNAi-expression cassette used for Lyc e 2- or GNTI-silencing; restriction sites are indicated (destroyed ones in brackets). (BHI: BamHI; B33: tuber/fruit-specific promoter; CCD: cross-reactive carbohydrate determinants; polyA: polyadenylation signal; 35S: constitutive promoter of Cauliflower Mosaic Virus).

To challenge the role of CCD-sIgE in plant food allergy, we chose two different RNA interference (RNAi) approaches to minimize CCD epitopes in plant-derived foodstuff: i) reduction of single glycoallergen Lyc e 2 in tomato, and ii) reduction of all CCD epitopes in tomato and potato (Figure 1A). The second approach employed silencing of N-acetylglucosaminyltransferase I (GNTI), an enzyme that catalyses the initial step of complex-type *N*-glycan formation in the Golgi apparatus and thus finally allows addition of *core* α1,3-fucose and β1,2-xylose in plants [29], [30], referred to as CCD epitopes (Figure 1B). Both RNAi approaches (Figure 1C, general construct design) intended to maintain in other respects natural allergen composition and offer the opportunity to minimize post-harvest treatment that may influence reactivity of peptide epitopes. Lack of α1,3-fucose and β1,2-xylose residues is tolerated well under standard growth conditions by both, Arabidopsis *complex glycan less1* (*cgl1*, At4g38240) null mutants [29], [31] and transgenic plants [30].

Importantly, by the chosen approach only *in-planta* synthesis of CCD epitopes was suppressed and not *N*-glycosylation *per se*, which is indispensable for correct protein folding [32] and plant vitality [33]. Therefore, RNAi-silenced tomato fruits and potato tubers could be compared to the wild-type situation with respect to sIgE and sIgG_4_ binding as well as basophil reactivity. Two independent patient groups with carbohydrate-specific IgE antibodies were investigated: one with confirmed potato and/or tomato allergy, and another with hymenoptera-venom allergy reporting no symptoms to the plant foods. The latter served as control for solely CCD-based reactions. To investigate possible benefits of CCD-reduced material for routine allergy testing, finally, plant extracts were biotinylated and coupled to streptavidin-ImmunoCAPs.

## Results

### Lyc e 2- and CCD-reduced plant lines are viable and gene-silencing is stable for several generations

**Figure 2:**
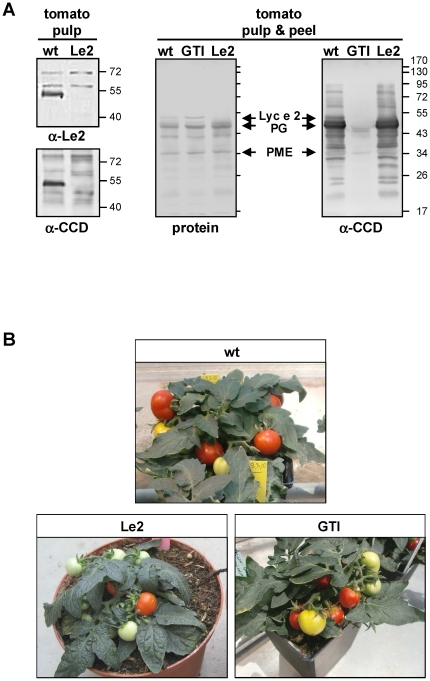
Verification of successful Lyc e 2- and GNTI-silencing in tomato. **A**: Immunoblots prepared with tomato fruit extracts of wild-type (wt), Lyc e 2-silenced (Le2), or GNTI-silenced (GTI) lines were developed either with α-Le2 or α-CCD polyclonal rabbit antiserum. Protein staining is shown as loading control for the blot developed with α-CCD. Note that the CCD pattern of Le2 is similar to wt, except for a faint band corresponding to Lyc e 2. Consistently with the immunoblots, enzymatic activity of vacuolar β-fructofuranosidase (invertase) was undetectable in Le2-fruit extracts (data not shown). Sizes of glycoprotein allergens are indicated: Lyc e 2 (∼52 kDa), PG (polygalacturonase 2A, 46 kDa), and PME (pectin(methyl)esterase, ∼35 kDa). **B**: Le2 and GTI tomato plants compared to wt. Note that both transformants are viable and form mature fruits.

For RNAi-mediated gene silencing of glycoallergen Lyc e 2 (β-fructofuranosidase or invertase, vacuolar isoform), initially a constitutive (35S) and a tuber/fruit-specific (B33) promoter were used (Figure 1C). Both promoters proved to be equally efficient in about 30% of the tomato transformants. As revealed by immunoblot analyses with rabbit antisera specific for either Lyc e 2 (α-Le2) or CCD (α-CCD), a glycoprotein of about 52 kDa is missing from tomato pulp tissue of Lyc e 2-silenced plants (further referred to as Le2, Figure 2A). However, since more than one band was recognized by α-Le2 in wild-type fruit extracts, proper designation of the missing band required additional analyses using peptide: N-glycosidase F (PNGase F) treatment (compare wt and Le2 *versus* a crossed Le2xGTI line, Figure S1 and Text S1). Notably, Le2 plants showed no striking phenotype in the greenhouse (Figure 2B), despite missing vacuolar β-fructofuranosidase activity (not shown).

In tomato, efficient general CCD reduction via GNTI-silencing was only achieved with the constitutive (35S) promoter. Out of 91 regenerated plants 6 (7%) displayed reduced CCD patterns. The two best lines carried CCD reduction beyond transformant generation T6. Immunoblot analyses conducted with α-CCD showed that whole fruit extracts of selected GNTI-silenced plants (further referred to as GTI) have clearly reduced CCD patterns (Figure 2A). Compared to wild-type, only faint recognition of most abundant glycoproteins remained, namely a double band around 45 kDa and a second glycoprotein of about 35 kDa (possibly pectin(methyl)esterase, PME). In the greenhouse, GTI plants were more susceptible to stray pathogen attack compared to tomato wild-type and Le2 plants, as already observed for corresponding Arabidopsis GNTI null-mutant *cgl1* [29]. Furthermore, directly stem-associated fruit parts turned necrotic during ripening (Figure 2B and Figure S1C), and especially fruits of older plants showed a patchy, yellow-red coloration.

**Figure 3:**
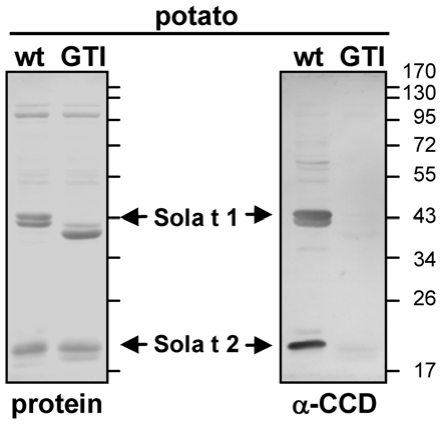
Verification of successful GNTI-silencing in potato. The immunoblot was prepared with extracts of wild-type (wt) and GNTI-silenced (GTI) tubers and developed with the CCD-specific rabbit antiserum (α-CCD). Sizes of known glycoprotein allergens Sola t 1 and Sola t 2 are indicated. The protein-stained blot confirms equal loading and reveals band shifts around 40–43 kDa in GTI extracts, likely due to different Sola t 1 (patatin) isoforms.

In potato, transformation was successful with both promoter constructs resulting in efficiently reduced CCD patterns. With the constitutive promoter, 28 out of 187 (15%) original transformants were strongly silenced, whereas with the tuber-specific promoter this was only the case for 5 out of 167 (3%) generated plants. Selected lines maintained low CCD levels during several vegetative reproduction cycles and showed no phenotype under greenhouse conditions, i.e. were indistinguishable from potato wild-type (not shown). Similarly to tomato, in CCD-reduced potato tubers only residual detection of abundant glycoproteins remained, most likely Sola t 1 (patatin of about 43 kDa) and Sola t 2 (cathepsin D-protease inhibitor of about 21 kDa) (Figure 3 and Figure S2B). From each tomato and potato set, best suppressed plants were chosen for further breeding and analyses.

### Le2 tomato fruit extracts activate basophils comparable to wild-type

**Figure 4:**
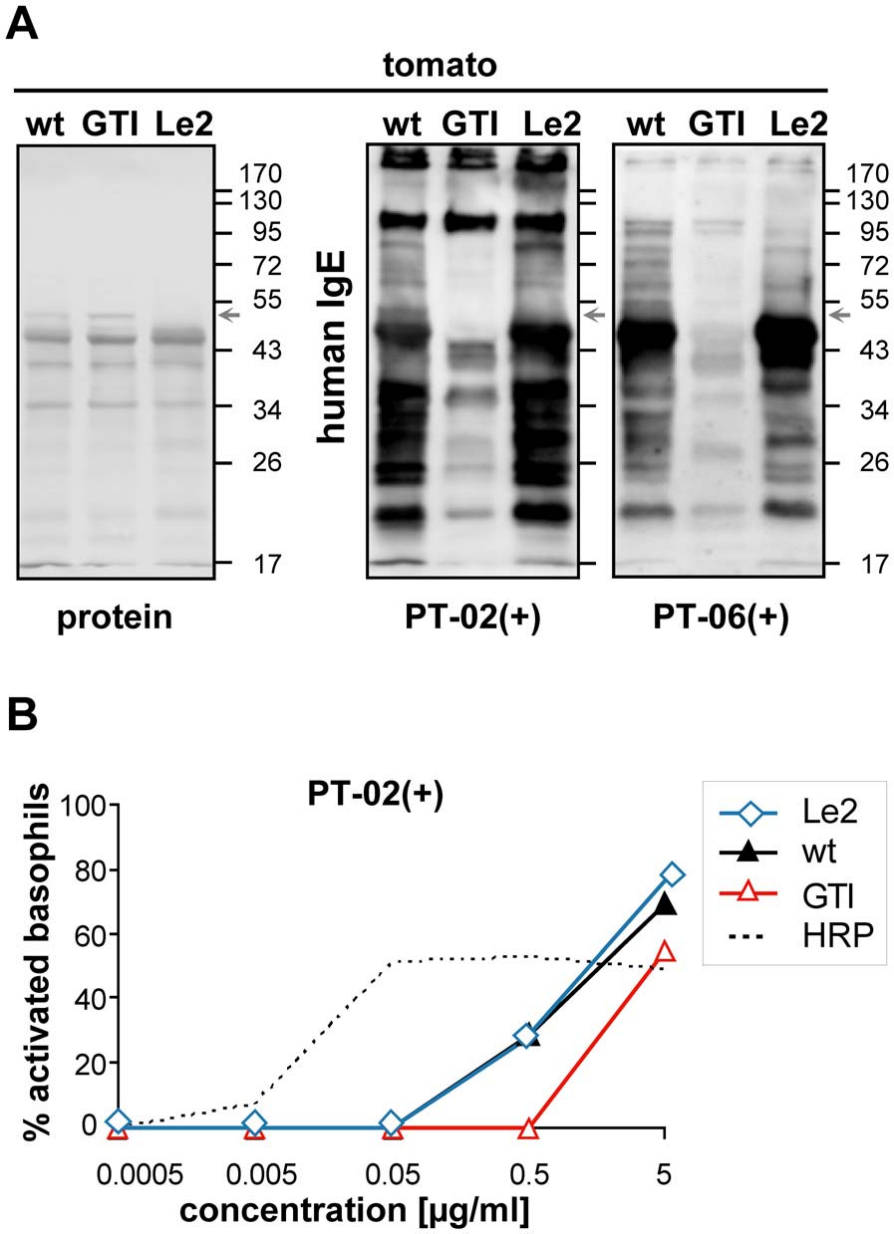
Lyc e 2 seems to be no key player among other CCD-bearing glycoproteins of tomato. **A**: Immunoblots prepared with whole fruit extracts (pulp with peel) of wild-type (wt), Le2, and GTI plants were developed with CCD-positive potato/tomato-allergic patient sera (PT-02(+) and PT-06(+)). Arrows point to a faint band around 52 kDa missing in Le2 (for Lyc e 2 size, compare Figure 2 and Figure S1). Protein staining is shown for equal loading. **B**: Basophil activation test of PT-02(+) with indicated tomato fruit extracts. Horseradish peroxidase (HRP), a vacuolar glycoprotein with plant-specific CCD epitopes, was used as control for CCD-dependent stimulation (dotted line).

Since Lyc e 2-reactivity was ascribed to CCD epitopes alone [7], [16] and 32 individuals with sIgE to tomato gave no signals with recombinant, unglycosylated Lyc e 2 on immunoblots (not shown), one aim of the present study was to investigate in CCD-positive patients what impact Lyc e 2 has among all tomato allergens. When blotted Le2 extracts of whole fruits (pulp with peel) were challenged with sera of CCD-positive patients (labeled (+), Table 1), Lyc e 2 reduction was detectable but less obvious, due to many other prominent glycoproteins of similar size (exemplarily shown for PT-02(+) and PT-06(+), Figure 4A, arrows). Interestingly, in CCD-positive food-allergic patients, basophil activation with Le2 extracts was comparable to wild-type (exemplarily shown for PT-02(+), Figure 4B).

**Table 1:** Allergic symptoms (patient history), specific and total IgE (ImmunoCAP analyses) of all patients investigated.

					Specific IgE (kU/l)										
		Allergic symptoms to			MUXF	HRP	YJV	HBV	Potato			Tomato			
	Patient	Potato	Tomato	HV	o214	o400	i3	i1	f35	wt	GTI	f25	wt	GTI	Total IgE (kU/l)
**CCD-negative**	**PT-03(−)**	CU (3)	OAS	–	0.11	0.18	0.66	1.12	0.29	0.58	0.52	1.21	1.05	0.73	541
	**PT-16(−)**	CU (1)	–	–	0	0	0.01	0.03	5.02	3.28	4.08	0.03	0.02	0.06	166
	**PT-20(−)**	–	OAS	–	0	0	0	0	0.16	0.04	0.04	2.20	0.35	0.35	162
	**PT-21(−)**	CU (3)	–	–	0.03	0.06	0.02	0.07	1.44	1.07	1.21	0.28	0.13	0.2	221
	**PT-23(−)**	CU (3)	OAS, dyspnoea	–	0	0	0	0	1.42‡	4.15‡	3.85‡	1.92‡	1.71‡	3.24‡	35.9
	**PT-26(−)**	–	OAS	–	0.19	0.24	0.11	0.20	0.57	0.39	0.34	2.08	0.80	0.56	77.4
	**PT-27(−)**	CU (1)	CU (1)	–	0.08	0.11	0.05	0.11	1.88	0.90	1.05	0.36	0.29	0.19	557
**CCD-positive**	**PT-01(+)** *	RC	CU (1)	–	0.20	0.62	0.66	1.90	3.93	1.20	1.06	3.76	0.29	0.91	552
	**PT-02(+)**	CU (1)	OAS	–	22.3	27.4	24.7	33.3	34.9‡	36.4‡	32.6‡	38.9‡	31.4‡	17.2‡	6840
	**PT-06(+)**	CU (1)	–	–	0.11	1.72	0.28	0.23	2.43	2.17	0.80	2.20	1.97	0.16	393
	**PT-09(+)** *	OAS; CU (1)	OAS	–	0.10	0.56	1.06	1.67	0.64	0.80	0.55	0.66	0.63	0.17	200
	**PT-22(+)**	–	GI	–	2.68	2.65	2.76	2.86	3.65	2.92	1.27	3.96	3.88	0.83	352
	**PT-34(+)**	–	OAS	–	2.44	0.49	0.11	0.45	4.17	1.43	0.84	3.55	4.23	0.35	332
	**PT-38(+)**	CU (1)	–	–	28.8	24.8	21.1	51.9#	35.8‡	34.5‡	18.0‡	25.4‡	27.6‡	3.74‡	1122
	**BW-28(+)**	–	–	systemic (YJV)	1.12	1.26	27.6	9.16	1.32	0.79	0.30	1.48	0.92	0.09	796
	**BW-32(+)**	–	–	systemic (YJV)	6.70	9.33	9.92	15.8	11.7	20.1	7.47	9.83	16.8	1.19	1000
	**BW-39(+)**	–	–	systemic (YJV)	1.07	2.28	14.8	3.13	1.74	1.02	0.83	1.79	0.9	0.29	68.5
	**BW-40(+)**	–	–	systemic (YJV)	7.51	4.55	63.3	11.3	5.34‡	6.52‡	3.72‡	4.40‡	5.51‡	0.98‡	737
	**BW-42(+)**	–	–	systemic (HBV)	1.40	3.05	2.18	9.52	2.19‡	2.71‡	1.94‡	2.21‡	2.35‡	0.69‡	41.6
	**BW-43(+)**	–	–	systemic (YJV)	2.77	1.61	>100	3.65	2.98	2.49	2.04	3.51	3.40	1.14	1610
**control**	**NA-05**	–	–	–	0	0	0	0	0.01	0.002	0	0.01	0.01	0.03	56.1
	**NA-16**	–	–	–	0	0	0.03	0.04	0.02	0.001	0.004	0.01	0.01	0.03	30.8
	**NA-18**	–	–	–	0	0	0.02	0.03	0	0	0	0	0.004	0.03	10.7

Allergic symptoms after contact with potato, tomato or hymenoptera venoms are listed according to patient history. Contact urticaria (CU, after direct contact with raw potato or tomato) was classified from 1 to 4 according to von Krogh and Maibach [51]. Total and specific IgE levels were measured with the UniCAP100 instrument (Phadia) using either commercial ImmunoCAPs for tomato (f25), potato (f35), HBV (i1), YJV (i3), HRP (o400), and MUXF (o214), or in-house-made ImmunoCAPs (wt and GTI). Specific IgE values ≥0.35 kU/l were considered positive.

*Borderline patients due to negative MUXF-sIgE and borderline positive HRP-sIgE levels.

# Recently stung by a honeybee but without allergic symptoms.

‡ For CCD inhibition with HRP, see Table 2. (BW: bee/wasp hymenoptera venom-allergic; CU: contact urticaria; GI: gastro-intestinal symptoms; GTI: GNTI-silenced; HBV: honeybee venom; HRP: horseradish peroxidase; HV: hymenoptera venom; MUXF: *N*-glycan structure according to the proglycan system (www.proglycan.com); NA: non-allergic control; OAS: oral allergy syndrome; PT: potato/tomato-allergic; RC: rhinoconjunctivitis; systemic: systemic reaction; wt: wild-type; YJV: yellow jacket venom).

### Selective loss of CCD epitopes in GTI plants

**Figure 5:**
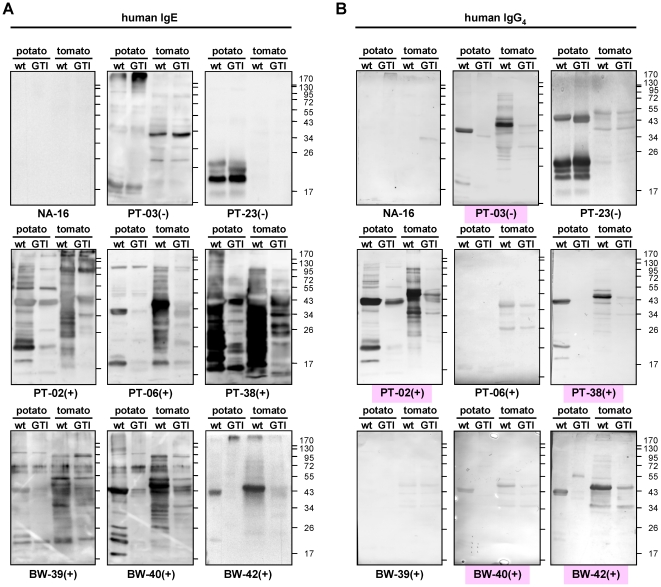
Blotted GTI extracts show reduced binding of CCD-positive patient sera and reveal differences in specific IgE and IgG_4_ patterns. **A**: Immunoblots of potato tuber and tomato fruit extracts (pulp with peel) were incubated with selected patient sera (NA: non-allergic control; PT: potato/tomato food-allergic; BW: bee/wasp hymenoptera venom-allergic) and developed for detection of human IgE. CCD-sIgE negative patients are labeled (−) and CCD-sIgE positive patients (+) (compare Table 1). **B**: After sensitive ECL detection of IgE binding, blots were additionally subjected to colorimetric development for visualizing bound IgG_4_. Sera that differentiate between wild-type (wt) and CCD-reduced (GTI) plant extracts at the IgG_4_ level are labeled pink.

As initially observed with the CCD-specific rabbit antiserum (Figure 2A and Figure 3), all patient sera with confirmed CCD-sIgE showed strongly reduced binding to GTI samples upon immunoblot analyses with wild-type and GNTI-silenced plant extracts (Figure 5A). However, there were no obvious differences between food- and hymenoptera venom-allergic patients. As expected, sera of non-allergic subjects (NA) did not show specific IgE binding and CCD-sIgE negative sera of potato/tomato-allergic control patients did not discriminate between wild-type and GTI (Figure 5A). Furthermore, blots of additionally investigated patient sera imply that known tomato and potato allergens are detectable in both, wild-type and GTI extracts to similar extent (independent of CCD epitopes, Figure S2).

Additional analyses of CCD-sIgG_4_ abundance also revealed clear discrimination between wild-type and CCD-reduced foodstuff for some patients (Figure 5B, marked pink), and moreover, unchanged peptide-specific binding. However, not all patients with CCD-sIgE also displayed CCD-sIgG_4_ and *vice versa* (Figure 5, compare panels A and B), particularly obvious for PT-03(−) (CCD-sIgE negative but CCD-sIgG_4_ positive), and PT-06(+) or BW-39(+) (CCD-sIgE positive but CCD-sIgG_4_ negative).

### Reduced basophil activation by GTI extracts in CCD-positive patients

To investigate the capability of CCD-sIgE to trigger effector-cell activation, and to elucidate whether presence of CCD-sIgG_4_ might have an influence, basophil-activation tests (BAT) with native plant food extracts or single plant glycoprotein horseradish peroxidase (HRP, routinely used to assess activation via CCD epitopes) were performed in a total of eight patients. Initially, three healthy non-allergic subjects were analyzed, and showed basophil activation with the positive control (α-IgE, ∼20%) but not with plant food extracts or HRP (data not shown).

**Figure 6:**
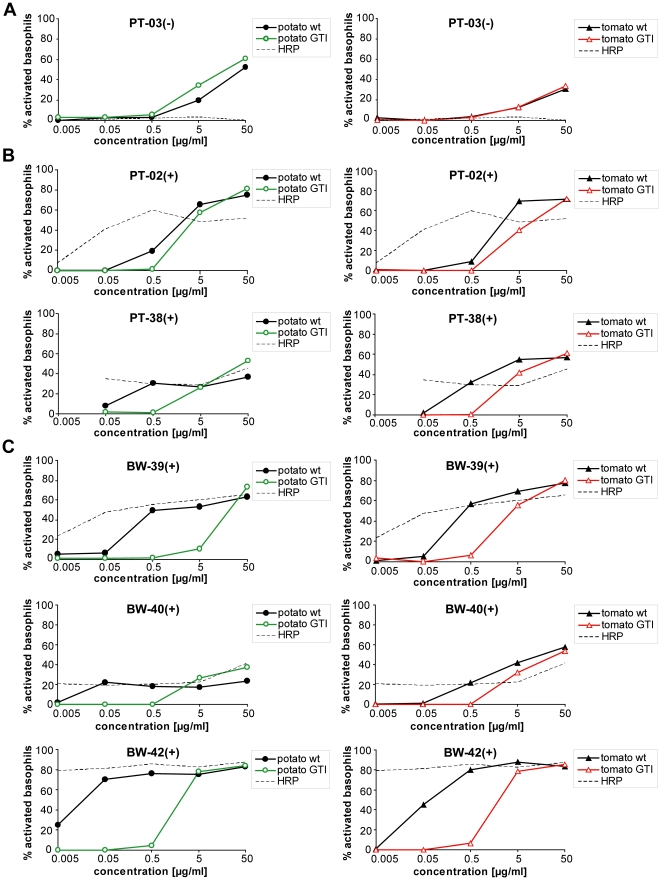
CCD-positive patients show decreased basophil activation with GTI extracts. Comparison of basophils stimulated with potato tuber (left panels) or tomato fruit extracts (right panels) of either wild-type (wt) or GTI plants. In all tests, horseradish peroxidase (HRP, dotted line) served as control for CCD-dependent stimulation. The percentage of activated basophils was calculated by subtracting values of spontaneous CD203c expression (negative control, PBS) from the values obtained with the particular allergen challenge. **A**: Potato/tomato-allergic patient without CCD-sIgE; **B**: Potato/tomato-allergic patients with CCD-sIgE; **C**: Hymenoptera venom-allergic patients with CCD-sIgE but no allergic symptoms to potato or tomato (for patient details, see Table 1).

Interestingly, basophils of all patients with confirmed sIgE to tomato or potato (Table 1) could be activated with wild-type plant food extracts, regardless whether symptoms were reported (potato/tomato-allergic group) or not (hymenoptera venom-allergic group) (Figure 6 and Figure S3). Activation by HRP was only observed for clear-cut CCD-positive patients (Figure 6B and C, dotted line), whereas patients without CCD-sIgE (PT-03(−), Figure 6A), or borderline CCD-positive potato/tomato-allergic patients (PT-01(+) and PT-09(+), Figure S3) revealed no activation upon HRP stimulation.

For patients without HRP response, stimulation with either wild-type or GTI extracts activated basophils similarly (Figure 6A and Figure S3). In contrast, basophils of HRP-responsive patients showed clearly reduced activation by GTI extracts. For PT-38(+), BAT results differed by up to one order of magnitude between wild-type and GTI extracts, whereas PT-02(+) showed weaker but detectable CCD discrimination (Figure 6B). Best discrimination was obtained with basophils of hymenoptera venom-allergic blood donors. In this CCD-positive patient group, stimulation differed by up to two orders of magnitude between wild-type and GTI extracts (Figure 6C). CCD-sIgG_4_ clearly present in some sera of the two CCD-positive patient groups (Figure 5B, marked pink), however, had no influence on the outcome of the BAT. For example, stimulation with potato and tomato wild-type extracts resulted in similarly strong basophil activation of BW-39(+) without CCD-sIgG_4_ and BW-42(+) with CCD-sIgG_4_ (compare Figure 6B and C).

### ImmunoCAP analyses reveal benefits of GTI extracts for allergy testing

**Figure 7:**
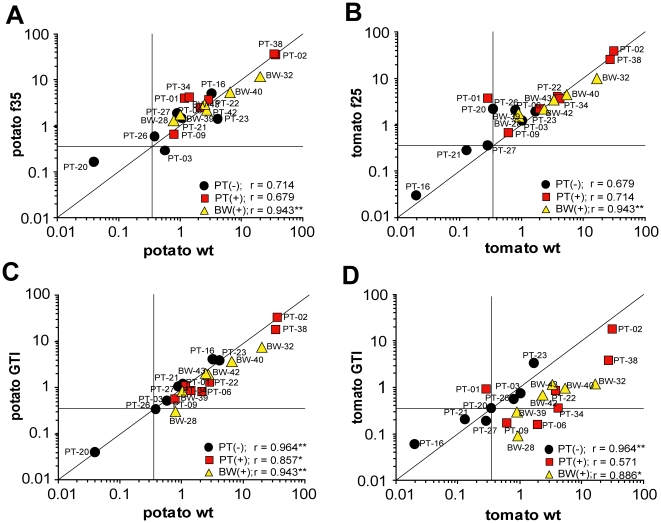
Correlation plots of specific IgE values determined with commercial and in-house-made ImmunoCAPs. Comparison of sIgE values in three different patient groups. **A**: Potato, commercial (f35) *versus* in-house-made wild-type (wt). **B**: Tomato, commercial (f25) *versus* in-house-made wt. **C**: Potato, in-house-made wt *versus* GTI. **D**: Tomato, in-house-made wt *versus* GTI. (Black circles: CCD-negative potato/tomato-allergic patients, red squares: CCD-positive potato/tomato-allergic patients, yellow triangles: CCD-positive hymenoptera venom-allergic patients). For sIgE values compare Table 1. Note that sIgE values of CCD-negative food-allergic patients (black circles) match more or less the bisecting line and those of CCD-positive patients (red squares and yellow triangles) shift downwards with GTI extracts (especially obvious for tomato). For better illustration, zero values were set to 0.01. Horizontal and vertical lines indicate the 0.35 kU/l threshold for sIgE positivity, bisecting lines congruency. Correlation coefficient r was calculated by the Spearman's rank correlation test, r = +1/−1 would be ideal (*: p<0.05; **: p<0.01).

To investigate feasibility of CCD-reduced foodstuff for allergy diagnosis, biotinylated wild-type and GTI extracts were coupled to streptavidin-ImmunoCAPs. For better comparison, sIgE values (Table 1) were plotted against each other or those obtained with commercial ImmunoCAPs (Figure 7), and additionally also against those obtained with HRP ImmunoCAP o400 (Figure S4 and Text S1). Specific IgE results determined with wild-type extracts were similar to those determined with commercial tomato (f25) and potato (f35) ImmunoCAPs (Table 1, Figure 7A and B). Furthermore, when analyzing sIgE results of wild-type and GTI ImmunoCAPs, sera of CCD-negative food-allergic patients (black circles) revealed no differences for potato and only slight differences for tomato (Figure 7C and D). By contrast, sIgE values of CCD-positive potato/tomato (PT, red squares) food-allergic and especially bee/wasp (BW, yellow triangles) hymenoptera venom-allergic patients were much lower with coupled GTI extracts as compared to wild-type (Figure 7C and D, more evident for tomato than potato), however not in all cases below the 0.35 kU/l (kilounits per liter) threshold.

### CCD inhibition reduces potato-specific IgE levels of one potato-allergic patient below threshold

To determine whether remaining sIgE recognition of GTI extracts by CCD-positive patient sera is due to residual CCD or to protein epitopes, we conducted inhibition experiments with HRP as CCD-competing glycoprotein. Initial dose-response tests identified 10 mg/ml HRP as sufficient for inhibiting CCD-sIgE (Figure S5A), and showed that in CCD-negative patient PT-23(−) sIgE recognition of tomato and potato is not affected (Table 2, Figure S5B). Five patient sera were investigated in detail, comprising two CCD-positive plant food-allergic and two hymenoptera venom-allergic patients (PT-02(+), PT-38(+), BW-40(+), BW-42(+); Table 2). HRP pre-incubation revealed that sIgE binding of CCD-positive sera to plant proteins can be inhibited to almost equal levels when comparing commercial, wild-type, and CCD-reduced ImmunoCAPs. This suggested that tomato and potato GTI extracts still contain residual CCD epitopes (compare immunoblot and BAT analyses), especially obvious for the two potato/tomato allergic patients with high CCD-sIgE levels (PT-02(+) and PT-38(+), Table 1). Sera of hymenoptera venom-allergic patients displayed inhibition below the 0.35 kU/l threshold, confirming that no peptide-sIgE antibodies to the plant food extracts are present. Surprisingly, the same was true for potato-allergic patient PT-38(+) using in-house-made potato wild-type and also commercial ImmunoCAP f35, albeit only at elevated inhibitor concentration (compare Table 2, Figure S5B), whereas potato/tomato-allergic patient PT-02(+) still displayed high sIgE levels after CCD inhibition for both plant foods.

**Table 2:** Specific IgE values of commercial and in-house-made ImmunoCAPs including HRP-mediated CCD inhibition for selected patients.

Patient	Potato						Tomato						Arabidopsis			
	f35		wt		GTI		f25		wt		GTI		wt		*cgl1*	
	−	+	−	+	−	+	−	+	−	+	−	+	−	+	−	+
**PT-23(−)**	1.37	1.40	1.41	1.24	1.18	1.07	2.23	2.11	0.84	0.86	2.36	2.42	0.10	0.10	0.06	0.06
**PT-02(+)**	41.2	2.37	31.5	3.98	21.4	2.67	47.2	4.13	30.6	3.11	14.6	3.53	28.6	3.31	4.83	2.73
**PT-38(+)**	34.8	0.42‡	31.8	0.26	7.88	0.09	30.8	0.21	23.3	0.17	3.71	0.09	18.9	0.28	0.67	0.20
**BW-40(+)**	5.90	0.10	5.31	0.05	1.71	0.02	5.05	0.05	4.01	0.11	1.05	0.05	3.55	0.13	0.26	0.09
**BW-42(+)**	2.61	0.04	2.09	0.01	1.06	0.01	2.54	0.03	1.75	0.08	0.66	0.07	1.74	0.09	0.11	0.08

CCD inhibition was done by incubating the respective patient sera over night at 4°C with HRP (10 mg/ml final concentration). Arabidopsis was included as control.

‡ When conducted with a final inhibitor concentration of 33.3 mg/ml HRP, the sIgE value decreased to 0.22 kU/l (below the 0.35 kU/l threshold, compare Figure S5B). (BW: bee/wasp hymenoptera venom-allergic; *cgl1*: *complex glycan-less* GNTI-null mutant; f25: commercial tomato ImmunoCAP; f35: commercial potato ImmunoCAP; GTI: GNTI-silenced; PT: potato/tomato-allergic; wt: wild-type).

As independent control, similar experiments were conducted with in-house-made ImmunoCAPs of Arabidopsis wild-type and GNTI-null mutant *cgl1*. All CCD-specific patient sera clearly discriminated between the two, but sIgE values of the two plant food-allergic patients did not lie below threshold with *cgl1*. Upon HRP inhibition, *cgl1*-sIgE values decreased below threshold only for PT-38(+) but lay clearly above threshold (2.73 kU/l) for PT-02(+).

## Discussion

Since protein- and carbohydrate-based allergic immune reactions are difficult to distinguish, this proof-of-principle study intended to explore the usefulness of selectively Lyc e 2- and CCD-reduced foodstuff. Importantly, our RNAi-based approaches represent more or less the physiological situation after contact with or ingestion of plant foods (i.e. challenge with an allergen mixture), in contrast to previous studies that reported histamine release assays conducted with only a single native, glycosylated allergen *versus* the recombinant, non-glycosylated allergen form [7], [14]–[16].

In this context it is noteworthy that tomato glycoallergen Lyc e 2, whose effector-cell triggering depends on CCD epitopes alone [7], [16], was no key player for reducing the allergenic potential of tomato fruits. An explanation for this finding is given by the recent study of Mertens and coworkers, revealing that CCD may induce basophil activation without clinical relevance in hymenoptera venom allergy [34]. We therefore doubt that Lyc e 2 is a true allergen, albeit inducing histamine release [7], [16] that appears to result from application of a single plant glycoprotein in high concentration, similar to HRP used as control in our BAT assays.

Compared to silencing only one CCD-bearing glycoprotein (Lyc e 2), GNTI silencing offered the possibility to remove essentially all CCD epitopes from the plant extracts investigated, without additional chemical treatment. In all clear-cut CCD-positive patients tested, BAT analyses conducted with CCD-reduced GTI foodstuff revealed diminished effector-cell triggering to various extents. This was especially obvious in the hymenoptera venom-allergic group reporting no symptoms to potato and/or tomato. Since analyses with CCD-negative patients verified that peptide epitopes are unaffected, biological activity of CCD-sIgE without obvious link to clinical symptoms was confirmed (compare [34]). Notably, decreased effector-cell stimulation detected with CCD-reduced GTI plant extracts lay in the same range as previously reported for basophil histamine-release assays obtained with extracts of either LTP-silenced (Lyc e 3, a true allergen) [35], [36] or profilin-silenced (Lyc e 1, a debated allergen [10]) [37] tomato plants, demonstrating equal relevance of CCD-sIgE and peptide-sIgE at this level.

Nevertheless, reasons for the apparent clinical insignificance of CCD-sIgE are not obvious and have been extensively discussed [reviewed in 2]. Low binding affinity of carbohydrate-specific IgE antibodies was previously ruled out [9]. Instead, to explain tolerance in allergy, it was hypothesized that some IgG fraction might function as CCD-blocking antibody [9], [38], [39]. If this assumption was correct, an influence on our BAT analyses would be expected, because whole blood was used that retains the individual balance between sIgE and sIgG_4_. However, results obtained with CCD-sIgG_4_ positive and negative patients revealed no effect of CCD-sIgG_4_ upon basophil activation with native potato or tomato wild-type extracts. Furthermore, despite clear IgE binding to wild-type plant food extracts, lack of clinical symptoms in the hymenoptera venom-allergic group was not accompanied by high sIgG_4_ levels. Therefore, blocking of CCD epitopes by CCD-sIgG_4_ antibodies can be probably ruled out.

The overall opinion that CCD-sIgE is irrelevant for triggering allergic symptoms and causes only ‘false-positive results’ in serum tests [2], [10] recently received support by a study in which plant-derived, CCD-decorated human lactoferrin did not elicit allergic symptoms among a limited number of patients (n = 3) upon double-blind placebo-controlled food challenge [12]. But remarkably our study identified one patient with symptoms to potato (PT-38(+)) as potential candidate for clinical relevance of CCD-sIgE, because after serum inhibition with HRP, potato-sIgE values lay below threshold. This finding is especially striking, since plant food-allergic patient PT-02(+) still displayed high sIgE levels after HRP inhibition (for both, tomato and potato), indicating presence of peptide-sIgE that would also explain the minimal differences observed in the BAT.

To assure that CCD epitopes may be needed occasionally to trigger allergic symptoms, identification of more patients like PT-38(+) is required to perform double-blind placebo-controlled challenges with cognate, essentially CCD-free plant material. However, as unsolved issue remains that stability and composition of plant-derived extracts is a general problem for routine *in-vitro* diagnosis [40], [41]. Thus, it is also possible that the causative potato allergen for PT-38(+) was missing from both, in-house-made and commercial ImmunoCAPs.

In order to improve routine testing for the majority of CCD-positive patients (i.e. those with clinically irrelevant CCD sensitization), GTI potato and tomato extracts were coupled to streptavidin-ImmunoCAPs. These in-house-made ImmunoCAPs resulted in clearly reduced sIgE values compared to the corresponding wild-types and commercial references, thus confirming the outcome of our initial immunoblot and BAT analyses. As an aside, commercial and streptavidin-ImmunoCAPs coupled with wild-type food extracts amounted to similar sIgE values, implying high quality of our in-house-made ImmunoCAPs. For selected patients with very high CCD-sIgE titers, HRP-inhibition experiments suggested that CCD reduction of the GNTI-silenced foodstuff might not be complete, especially in potato. However, HRP inhibition also reduced sIgE binding to Arabidopsis GNTI-null mutant *cgl1* (definitely lacking CCD [42]), indicating that some IgE antibodies also bound to the HRP backbone. This alternatively might explain the under-threshold potato-sIgE values of PT-38(+) observed upon HRP inhibition. By contrast, PT-02(+) still showed strong residual sIgE binding to *cgl1* after HRP inhibition, pointing to recognition of additional peptide epitope(s) in leaf extracts. Since PT-02(+) displays symptoms to diverse pollens and other plant sources, this finding is interesting but not that surprising.

As inferred from another study on tobacco GTI-antisense plants lacking measurable GNTI activity despite almost wild-type-like CCD patterns on immunoblots [43], GNTI is likely suppressed below detection limits in our potato and tomato GTI-RNAi lines. CCD-reduced foodstuff therefore provides the possibility to improve allergy testing with whole extracts. Furthermore, such CCD-reduced plants also offer the opportunity of heterologous protein expression, especially when post-translational modification without perturbation by CCD epitopes is required. Despite availability of GNTI-null mutant *cgl1*, Arabidopsis - as a small weed - is not suitable for high yield applications. Also, the protein pattern of leaves is much more complex (than e.g. of seeds, fruits, or tubers), which could interfere with protein purification.

In summary, the GNTI-silenced tomato and potato lines described in this study proved to be a valuable tool for evaluating contribution of CCD- *versus* peptide-specific determinants to food-allergic reactions. We confirmed that for most patients investigated (except PT-38(+)) presence of CCD-sIgE is clinically irrelevant. Hence, the described approach bears the potential to improve existing diagnostic tools (BAT and sIgE determination). Since phenotypic deviations are negligible, CCD-reduced plants likely constitute an ideal expression system for glycosylated allergens. Thus, they should be perfectly suited for state-of-the-art component-resolved allergy diagnosis in the near future.

## Materials and Methods

### Ethics Statement

The study protocol was approved by the ethics committee of the Institutional Review Board of Münster University, School of Medicine (Permit no. 2007-451-f-S, ‘Investigations concerning CCD epitopes of allergenic glycoproteins’). Blood samples (50 ml per donor) were obtained under written informed consent and used for immunoblot development, basophil-activation tests, and ImmunoCAP analyses.

### Tomato and potato RNAi transformants, Arabidopsis *cgl1* mutant

For creation of tomato and potato RNAi transformants, total RNA was purified from tomato (*Lycopersicon esculentum* cv. Moneymaker ‘Micro-Tom’) or potato (*Solanum tuberosum* cv. Désirée) leaf tissue. GNTI-specific cDNA sequences were amplified for each cultivar using oligo-dT-primed reverse transcription with primers 5′-N_6_- GTCGAC CAA TTA AGG GCT CTT GTT C-3′ and 5′-N_6_- GGATCCGG CCA CTT TGG AG- 3′. Similarly, Lyc e 2-specific cDNA was obtained from tomato-fruit RNA using primers 5′-N_3_- GTCGACA CGA AGA GTA CTG TGG GGA TG-3′ and 5′-N_3_- GGATCCCT AGT TTG ATC AGC ACA GAA GTG AGT CTC-3′ (SalI and BamHI sites underlined). The resulting fragments were cloned in vector pUC-RNAi [44], and introduced into binary plant-expression vectors, driven either by a constitutive (35S) [45] or a tuber/fruit-specific (B33) [44] promoter (Figure 1C). Binary constructs were introduced into Agrobacteria and used to transform the corresponding plant cultivars [46], [47]. Arabidopsis GTI-null mutant *cgl1-3* (described in Frank et al.) [42] served as control for ImmunoCAP analyses.

### Preparation of protein extracts

Fresh tomato fruits without seeds or potato tubers were cut into small pieces and ground in liquid nitrogen to yield fine powder. For SDS-PAGE and immunoblot analyses, frozen powder was extracted with ice-cold buffer (50 mM of HEPES-NaOH pH 8, 250 mM NaCl, 2 mM Na_2_S_2_O_5_, 1 mM EDTA), 1 mM Pefabloc SC (Serva, Heidelberg, Germany), and Polyvinylpolypyrrolidone (0.1 mg/ml, Sigma-Aldrich, Taufkirchen, Germany) to prevent protein oxidation. For basophil activation tests (BAT), frozen powder was extracted with phosphate-buffered saline (PBS; pH 8 for tomato and pH 7.4 for potato), supplemented with 1 mM Pefabloc SC and processed as above. For preparation of in-house-made ImmunoCAPs, extraction of potato tubers was done as described for BAT. Due to low pH and protein contents, tomato fruit extracts were prepared with ‘high’ PBS (200 mM Na_2_HPO_4_ pH 9, 250 mM NaCl, 2 mM Na_2_S_2_O_5_, 1 mM EDTA, and 0.05% (v/v) Triton-X100) and supplements as above. Extraction of Arabidopsis leaves was done with last mentioned buffer. Protein contents were determined with Bradford reagent (Bio-Rad, Munich, Germany) and bovine serum albumin (BSA) as standard protein. Aliquots were stored at −80°C until use.

### Preparation of recombinant Lyc e 2 antigen for rabbit immunization

The coding sequence of mature vacuolar β-fructofuranosidase (Lyc e 2) [48] was used for *E. coli*-based expression and purification of recombinant protein. RT-PCR was conducted with tomato (*Lycopersicon esculentum* cv. Moneymaker “Micro-Tom”) fruit RNA using primers 5′-N_3_- CATATG TAT GCG TGG TCC AAT GCT ATG CTT AG-3′ and 5′-N_3_- GGATCCTT ACA AGT CTT GCA AAG GAA GGA TTG-3′ (NdeI and BamHI sites underlined). Amplified cDNA fragments were inserted into vector pET16b, allowing for overexpression in *E. coli* BL21(DE3):pLysS cells (Novagen/Merck, Darmstadt, Germany). Recombinant Lyc e 2 protein with His-tag was isolated with Ni-NTA agarose (Qiagen, Hilden, Germany) under denaturing conditions. This fraction was used in a pre-study with sera of subjects showing reactivity to tomato extracts, and for production of a polypeptide-specific polyclonal rabbit antiserum (α-Le2; Eurogentec, Seraing, Belgium).

### Immunoblot analyses

Prior to blot development, protein extracts (20 µg for tomato, 12 µg for potato) were separated by SDS-PAGE, blotted to nitrocellulose and stained with Ponceau S (Serva) as described previously [42]. For Lyc e 2 detection, the polypeptide-specific α-Le2 antiserum (described above) served as first antibody (1∶5,000 in 2% skimmed milk/2× Tris-buffered saline (TBS) with 0.1% [v/v] Tween-20 (TBST) for 2 hours) and HRP-labeled goat anti-rabbit IgG conjugate (Bio-Rad) as second antibody (1∶10,000 in 2% skimmed milk/TBST for 1 hour). For detection of CCD epitopes, a polyclonal rabbit anti-HRP serum (α-CCD, Sigma-Aldrich, Taufkirchen, Germany) was used as first antibody (1∶10,000 in 2% skimmed milk/2× TBST for 2 hours) followed by the second antibody (as above). For detection of sIgE, blots were first incubated with patient sera (1∶10 in 2% skimmed milk/TBST for 3 hours) followed by HRP-labeled affinity-purified goat antibodies to human IgE (KPL, Gaithersburg, Maryland, USA; 1∶10,000 in TBST for 1 hour). Signals were visualized with the ECL-Advance Western-Blot Detection Kit (Amersham/GE Healthcare, Freiburg, Germany). After chemiluminescent IgE detection, bound IgG_4_ was visualized via alkaline phosphatase (AP)-conjugated mouse anti-human IgG_4_ monoclonal antibodies (BD Biosciences; Heidelberg, Germany; 1∶500 in TBST for 1 hour) using colorimetric AP substrates (Promega, Mannheim, Germany).

### Patient selection and sera characterization

Two patient groups were recruited separately according to patient history and specific IgE levels determined by ImmunoCAP analyses (a routine *in-vitro* method used for allergy diagnosis): the potato and/or tomato allergic group comprised initially 26 patients with reported symptoms. Due to negative potato- and tomato-sIgE 12 patients were subsequently excluded. Of the remaining 14 patients seven displayed CCD-sIgE to HRP and MUXF (isolated *N*-glycan chains of bromelain). The others served as CCD-negative control. The second group comprised hymenoptera venom-allergic patients with confirmed sIgE towards CCD, but without reported symptoms to potato and tomato. CCD-sIgE negative patients are labeled (−) and CCD-sIgE positive patients (+) (compare Table 1). Additionally, three healthy subjects without history of allergic reactions and confirmed absence of sIgE to potato, tomato, hymenoptera venoms, HRP, and MUXF served as non-allergic controls (for an overview of investigated patients, see Table 1).

### Basophil-activation test (BAT)

BAT was performed as described earlier by Mertens et al. [34]. In brief, heparinized whole blood was incubated for 15 min at 37°C with 10-fold serial dilutions of the allergen extracts in PBS ranging from 50 to 0.005 or 5 to 0.0005 µg/ml. To determine reactivity towards plant-derived CCD, HRP was included in the same concentration range. To confirm cell responsiveness, 0.2 µg of a monoclonal anti-IgE antibody (clone BE5; EurobioSciences, Friesoythe, Germany) served as CCD-independent positive control. After stimulation, the reaction was stopped by addition of 20 mM EDTA in PBS and centrifugation at 400×g. Basophils were stained with 10 µl anti-CD203c-PE (Beckman-Coulter, Krefeld, Germany) for 45 min at room temperature in the dark. Erythrocytes were destroyed using ‘whole blood lysing reagent’ (Beckman-Coulter). After washing and resuspending the cells in PBS with 1% BSA, a total of 60,000 cells were measured using the FACSCalibur flow cytometer equipped with CellQuestPro software (BD Biosciences) and subsequently the percentage of activated cells was determined.

### Preparation of in-house-made ImmunoCAPs

In-house-made ImmunoCAPs were prepared by coupling biotinylated plant extracts to streptavidin-ImmunoCAPs (o212, Phadia, Freiburg, Germany). Coupling followed basically previous protocols [49], [50]. In brief, biotinylation was performed using the EZ-Link® Sulfo-NHS-Biotinylation Kit (PIERCE, Rockford, IL, USA) at pH 9 for 30 min at room temperature in 2.5-fold molar excess based on major proteins. To remove excess biotin, desalting spin columns were used, equilibrated with PBS (pH 8) and 0.05% Triton-X100. For coupling, 50 µl of each biotinylated extract were added to a streptavidin-CAP and incubated for 30 min in the UniCAP100 instrument (Phadia) before performing specific IgE assays (described below).

### ImmunoCAP analyses

Total and specific IgE levels were measured with UniCAP100 and corresponding kits (Phadia) using either commercial ImmunoCAPs for tomato (f25), potato (f35), hymenoptera venoms (i1 and i3), HRP (o400), and MUXF (o214), or the in-house-made ImmunoCAPs. Specific IgE values ≥0.35 kU/l were considered positive. Correlations between commercial and in-house-made ImmunoCAPs were statistically analyzed using the Spearman's rank correlation test and SPSS version 15.0 (SPSS Inc., Chicago, IL, USA). For CCD inhibition, the optimal HRP concentration was determined in a pre-study using 0.67 to 33.3 mg/ml HRP (final concentration) and over-night incubation with patient sera at 4°C prior to sIgE determination with commercial HRP ImmunoCAP o400. A final concentration of 10 mg/ml was found to be sufficient and used for further analyses.

## Supporting Information

Text S1 Supporting Methods, Results & Discussion, References, and Figure Legends.
(DOC)

Figure S1 PNGase-F treatment of tomato fruit extracts verifies efficient Lyc e 2-silencing.
(TIFF)

Figure S2 GNTI-silenced plants maintain CCD-independent specific IgE and IgG_4_ binding.
(TIFF)

Figure S3 Basophil activation in borderline CCD-positive potato/tomato-allergic patients.
(TIFF)

Figure S4 Correlation plots of specific IgE values determined with commercial and in-house-made ImmunoCAPs.
(TIFF)

Figure S5 Inhibition of CCD-specific IgE binding by horseradish peroxidase (HRP).
(TIFF)
