# Supplementary material for: Reduction of Cross-Reactive Carbohydrate Determinants in Plant Foodstuff: Elucidation of Clinical Relevance and Implications for Allergy Diagnosis
Source: PLoS One. 2011 Mar 14;6(3):e17800. doi: 10.1371/journal.pone.0017800 (PMC3056789; doi:10.1371/journal.pone.0017800)
Supplement: Text S1 — Supporting Methods, Results & Discussion, References, and Figure Legends. (DOC) [file pone.0017800.s001.doc]

**Text S1**

**Supporting Methods**

**Peptide: N-glycosidase F treatment for identification of Lyc e 2 bands**

For peptide: N-glycosidase F (PNGase F) treatment, tomato pulp tissue was extracted with 100 mM HEPES-NaOH pH 8, supplemented with 500 mM NaCl, 2 mM Pefabloc SC (Serva, Heidel­berg, Germany), and poly­vinyl­poly­pyrrolidone (PVPP, Sigma-Aldrich, Tauf­kirchen, Germany) to prevent protein oxidation. Soluble proteins (30 µg) were boiled in the presence of 0.1% SDS (w/v, final concentration) for 3-5 min to enhance accessibility of *N*‑glycans. Heated samples were supplemented with 0.5% Triton X-100 (v/v, final con­cen­tra­tion), 10 mM EDTA and 1% β-mercaptoethanol before adding 0.5 U of peptide: N‑glycosidase F (PNGase F, Roche, Mannheim, Germany). After incubation at 37°C for 20 h, samples were separated by 11% SDS-PAGE, blotted to nitrocellulose and stained for protein prior to blot development. Mock-incubated tomato extracts served as negative (-) control. PNGase F treatment releases *N*‑glycans that lack α1,3-fucoses [Tretter et al., 1991] and thus reduces binding of Jack Bean lectin Concanavalin A (ConA) to GTI extracts [29,30], as revealed by peroxidase-coupled affinoblot development in the presence of Ca^2+^ and Mn^2+^ ions [Faye and Chrispeels, 1985].

**Preparation of blot membranes for additional antibody probing**

For probing with succeeding antibodies, blots were incubated in blot-strip buffer (2% SDS, 0.7% β-mercaptoethanol in 62.5 mM Tris pH 6.7; ECL-Avance Western-Blot Detection Kit manual (Amersham/GE Healthcare, Freiburg, Germany) at 50°C for 30 min followed by extensive washing with TBST.

**Supporting Results & Discussion**

**Identification of Lyc e 2 on immunoblots**

To verify CCD reduction in GNTI-silenced (GTI) tomato plants and to confirm the missing protein in Lyc e 2-silenced fruits identified by the α-Le2 antiserum as Lyc e 2 (vacuolar β‑fructo­furanosidase), tomato extracts were treated with peptide: N-glycosidase F (PNGase F). This endoenzyme can only release *N*-glycans from asparagine, when α1,3-fucose is missing ([Tretter et al., 1991]; compare Figure S1A). To enable cleavage of *N*-glycans in Le2, they were crossed with GTI plants resulting in dually silenced Le2xGTI lines (Figure S1). Immunoblots developed with the α-CCD serum revealed clear reduction of CCD epitopes in fruit extracts of GTI and Le2xGTI compared to wild-type (Figure S1B). By contrast to α‑CCD, ConA labels predominantly terminal mannoses of high mannose- and bisected hybrid-type glycopeptides compared to the central tri-mannosyl region of complex-type glyco­peptides [Brewer and Bhattacharyya, 1986], and thus bound to *N*-glycans of GTI and Le2xGTI (bisected hybrid-type without α1,3-fucose residues) but not wild-type (complex-type *N*-glycans with α1,3-fucose residues; Figure S1A and B). In PNGase F-treated GTI and Le2xGTI extracts, loss of ConA binding was observed, verifying successful *N*-glycan release by PNGase F. Blot development with α-Le2 revealed binding to two proteins at 52 kDa and 56 kDa. Miron et al. (2002) described Lyc e 2 as 52 kDa and smaller (probably degradation products). Where­as the 52-kDa protein was recognized by α-CCD in wild-type (complex *N‑*glycans) and by ConA in GTI (mannose-terminated, bisected *N*-glycans), the 56-kDa protein was not bound by either of the two. Since described as glycoprotein solely decorated with CCD epitopes [16], only the 52-kDa band can be Lyc e 2. Furthermore, the size shift of ~5 kDa after PNGase F treatment observed in GTI extracts (marked by red arrowheads) is in good agreement with use of all four *N*-glycosylation sites, accounting for ~1.34 kDa each. In contrast, *N*-glycan(s) of the 56-kDa band are already released by PNGase F in wild-type (shifting to the height of fully glycosylated Lyc e 2, marked with blue asterisks), identifying the 56‑kDa band as a glyco­protein lacking α1,3-fucose residues (different β-fructo­furano­si­dase isoform?).

**ImmunoCAP analyses with wild-type potato and tomato extracts versus HRP**

Similar to the analyses of sIgE results obtained with in-house-made *versus* commercial ImmunoCAPs, values of wild-type and CCD-reduced ImmunCAPs were also plotted against HRP (o400) (Figure S4). Wild-type *versus* HRP-sIgE values of CCD-positive patients (red squares and yellow triangles) matched more or less the bisecting line (by contrast to CCD-negative patients; Figure S4A and B, black circles), and shifted when HRP was plotted against CCD-reduced GTI extracts (Figure S4C and D; more obvious for tomato than potato). This demonstrates that in both CCD-positive patient groups, CCD-sIgE accounts for major recognition of potato and tomato, and confirms that CCD-reduced plant extracts would improve allergy testing, assuming that CCD-sIgE is clinically irrelevant.

**Supporting References**

Brewer CF, Bhattacharyya L (1986) [Specificity of concanavalin A binding to asparagine-linked glycopeptides. A nuclear magnetic relaxation dispersion study.](http://www.ncbi.nlm.nih.gov/pubmed/3711088) J Biol Chem 261: 7306-7310.

Faye L, Chrispeels MJ (1985) Characterization of N-linked oligosaccharides by affinoblotting with Concanavalin A-peroxidase and treatment of the blots with glycosidases. Anal Biochem 149: 218–244.

Gruden K, Strukelj B, Ravnikar M, Poljsak-Prijatelj M, Mavric I, et al. (1997) Potato cysteine proteinase inhibitor gene family: molecular cloning, characterisation and immune­cyto­chemical localisation studies. Plant Mol Biol. 34: 317-323.

Miron D, Petreikov M, Carmi N, Shen S, Levin I, et al. (2002) Sucrose uptake, invertase localization and gene expression in developing fruit of *Lycopersicon esculentum* and the sucrose-accumulating *Lycopersicon hirsutum*. Physiol Plant 115: 35-47.

Tretter V, Altmann F, März L (1991) Peptide-N4-(N-acetyl-beta-glucosaminyl)asparagine amidase F cannot release glycans with fucose attached alpha 1-3 to the asparagine-linked N‑acetylglucosamine residue. Eur J Biochem 199: 647-652.

Vandevenne E, Van Buggenhout S, Duvetter T, Brouwers E, Declerck PJ, et al. (2009) Development and evaluation of monoclonal antibodies as probes to assess the differences between two tomato pectin methylesterase isoenzymes. J Immunol Methods. 349: 18-27.

Westphal S, Kempf W, Foetisch K, Retzek M, Vieths S, et al. (2004) Tomato profilin Lyc e 1: IgE cross-reactivity and allergenic potency. Allergy 59: 526-32.

Willerroider M, Fuchs H, Ballmer-Weber BK, Focke M, Susani M, et al. (2003) Cloning and molecular and immunological characterisation of two new food allergens, Cap a 2 and Lyc e 1, profilins from bell pepper (*Capsicum annuum*) and Tomato (*Lycopersicon esculentum*). Int Arch Allergy Immunol 131: 245-55.

**Supporting Figure Legends**

**Figure S1. PNGase-F treatment of tomato fruit extracts verifies efficient Lyc e 2-silencing. A**: *N*‑glycan structures according to the proglycan system (www.proglycan.com) with indication of PNGase F release when α1,3-fucose is missing [Tretter et al., 1991]. **B**: Immuno­blot analyses of untreated (‑) and PNGase F-treated (+) tomato fruit extracts of wt, GTI, and Le2xGTI (crossed line). To enable comparison of protein-stained bands with those detected by antibodies or ConA, two dominant bands were marked with a water resistant pen (upper black dots in GTI lanes appear void in α‑Le2 and ConA developed blots). Note that upon *N*-glycan removal by PNGase F, a band stained by α‑Le2 shifts ~5 kDa in the GTI extract corresponding to use of all four potential *N*‑glyco­sylation sites in Lyc e 2 (~1.34 kDa each). Since in the Le2xGTI extract no comparable shift occurs, Lyc e 2 is supposedly absent. Red arrowheads mark Lyc e 2 positions and blue asterisks an un­identified protein also recognized by α‑Le2, but with different charac­teristics (related β-fructofuranosidase isoform?). **C**: Photographs of tomato wt, GTI, and crossed line Le2xGTI. All transformed plants are viable and produce mature, almost red fruits. In the green­house, Le2xGTI lines performed similarly to GTI (compare Figure 2B). (α-CCD: CCD-specific rabbit antiserum; α-Le2: Lyc e 2 (polypeptide)-specific rabbit antiserum; ConA: Con­cana­valin A (Jack Bean lectin, pre­dominantly labeling terminal mannoses of high mannose- and bisected hybrid-type glyco­peptides).

**Figure S2. GNTI-silenced plants maintain CCD-independent specific IgE and IgG_4_ binding.** Blots prepared with tomato fruit (A) and potato tuber (B) extracts of wild-type (wt) and GNTI-silenced (GTI) plants were incubated with selected CCD-sIgE negative (-) or positive (+) patient sera, and developed first for sIgE, followed by detection of sIgG_4_ (compare Table 1, Figure 5). Equal protein loading is shown on the left. Protein bands marked **a-n** probably represent the following tomato and potato allergens. **A**: Tomato extracts. **a**) Lyc e 2 (vacuolar β-fructo­furanosi­dase, ~50-52 kDa [16,18,27]) carrying four *N*‑glycans (compare Figure S1) is only recognized by CCD-sIgE or sIgG_4_. **b**) Poly­galacturonase 2A (PG, 46 kDa) with four potential *N*-glycosylation sites [18,19] is also recognized in GTI. Besides, binding to another protein, possibly an isoform of PG or Peroxidase1 (pathogenesis-related protein, 45 kDa, with 7 potential *N*-glyco­­sylation sites [28]) is observed. **c**) Pectin­(methyl)­esterase (14 kDa [18] or 34-36 kDa [19], Vandevenne et al., 2009) is recognized in wt and GTI by sIgE of PT-09(+). **d**) An unidentified protein of about 22-24 kDa is recognized by sIgG_4_ of PT-09(+). Although bands recognized in wt and GTI do not migrate at equal height, apparently the same protein epitope is recognized (compare also size shifts observed for Sola t 1 [h] in B). **e**) Lyc e 1 (profilin, 14‑16 kDa, without *N*-glycans) [18,27, Willer­roider et al., 2003, Westphal et al., 2004] is recognized in wt and GTI by sIgE and sIgG_4_ of PT-22(+). **f**) Lyc e 3 (Lipid-Transfer-Protein LTP, 9 kDa, without *N*-glycans) [36] is recog­nized in wt and GTI by sIgE and sIgG_4_ of PT-22(+). **B**: Potato extracts. **g**) An unidentified protein of about 100 kDa, also visible in the protein-stained blot, is labeled in wt and GTI by sIgE and to lesser extent by sIgG_4_ of PT-20(-). **h**) Sola t 1 (patatin, a glycoprotein of about 40-43 kDa) [21-25]. Note that the band shift is already visible in the protein-stained blot. Since both, wt and GTI-shifted Sola t 1 forms are recognized similarly by sIgE and sIgG_4_ of PT-20(-), and by sIgG_4_ of PT-23(-), CCD-independent peptide epitopes appear unchanged. **i**) Sola t 2 (cathepsin D-protease inhibitor, a glycoprotein of about 21 kDa) [17]. **j**) Sola t 3 (cysteine-protease inhibitor, a protein of 21 kDa without *N*-glycans) [Gruden et al., 1997, 17]. IgE and IgG_4_ development of PT-20(-) and PT-23(-) showed multiple bands in the 20‑kDa range. Several interpretations are possible: Sola t 2 exists in at least two forms (compare Sola t 1 [h]), because the most intense band recognized by sIgG_4_ of PT-20(-) shifts in GTI compared to wt. This was also observed with sIgE of PT-23(-). Sola t 2 seems to be less abundant in potato tubers, because no difference between wt and GTI at ~20 kDa is visible in the protein-stained blot. Instead, the band most likely represents Sola t 3, which is similarly recognized by sIgG_4_ of PT-23(-) in GTI and wt. **k**) An unidentified protein of about 19 kDa, binding only anti­bodies of PT‑23(-) but not PT‑20(-). **m**) Sola t 4 (aspartic-protease inhibitor, consisting of two subunits (16 + 4 kDa) without *N*-glycans) [17] is recog­nized in wt and GTI by sIgE and sIgG_4_ of PT-20(-) and PT-23(-). **n**) An un­identified protein of about 9 kDa without cross-reaction to tomato proteins (of similar molecular weight range in presented patients, data not shown).

**Figure S3**. **Basophil activation in borderline CCD-positive potato/tomato-allergic patients. A**: Blots prepared with potato tuber and tomato fruit extracts of wild-type (wt) and GNTI-silenced (GTI) plants were incubated with sera of PT-01(+) or PT-09(+) (for patient details see Table 1) and first developed for bound IgE, followed by detection of bound IgG_4_ (compare Figure 5). **B**: The BAT with patient sera (mentioned in A) was conducted with wt and CCD-reduced (GTI) potato tuber (left panels) or corresponding tomato fruit extracts (right panels). Note that stimulation with horseradish peroxidase (HRP, dotted line) did not result in basophil activation, although CAP results were borderline positive with HRP and negative with MUXF (Table 1).

**Figure S4: Correlation plots of specific IgE values determined with commercial and in‑house-made ImmunoCAPs.** Comparison of sIgE values in three different patient groups. **A:** HRP (o400) *versus* in-house-made potato wild-type (wt). **B**: HRP (o400) *versus* in-house-made tomato wt. **C**: HRP (o400), *versus* in-house-made GNTI-silenced potato (GTI). **D**: HRP (o400) *versus* in-house-made tomato GTI. (Black circles: CCD-negative potato/tomato-allergic patients, red squares: CCD-positive potato/tomato-allergic patients, yellow triangles: CCD-positive hymenoptera venom-allergic patients). For sIgE values compare Table 1. Note that for wt potato and tomato extracts sIgE values of both CCD-positive patient groups (red squares and yellow triangles) correlate with HRP but those of CCD-negative food-allergic patients (black circles) do not, because HRP levels are below threshold. As in Figure 7, sIgE values of CCD-positive patients shift downwards with GTI extracts (especially obvious for tomato). For better illustration, zero values were set to 0.01. Horizontal and vertical lines indicate the 0.35 kU/l threshold for sIgE positivity, bisecting lines congruency. Correlation coefficient r was calculated by the Spearman’s rank correlation test, r = +1/-1 would be ideal (*: p<0.05; **: p<0.01).

**Figure S5: Inhibition of CCD-specific IgE binding by horse­radish peroxidase (HRP).** CCD inhibition was conducted by incubating patient sera with or without (control) vacuolar plant glycoprotein HRP (0.67 to 33.3 mg/ml final concentration) over night at 4°C. Specific IgE values were determined with commercial ImmunoCAPs. **A:** HRP (o400). For CCD-positive potato/tomato allergic patients PT‑02(+) and PT-38(+), inhibitor concentrations ≥10 mg/ml reduced HRP-specific IgE values below the 0.35 kU/l threshold (horizontal line) indicative of complete CCD inhibition. **B:** Potato (f35-CAP). CCD inhibition lowered potato-specific IgE values for both CCD-positive patients, however, below threshold only for PT‑38(+) but not PT-02(+). This indicates either potential clinical relevance of CCD-specific IgE in PT-38(+) or missing of the causative potato allergen from commercial ImmunoCAP f35. Note that sIgE values of CCD-negative patient PT-23(-) are not affected.
