# Supplementary figures and images for: Reduction of Cross-Reactive Carbohydrate Determinants in Plant Foodstuff: Elucidation of Clinical Relevance and Implications for Allergy Diagnosis
Source: PLoS One. 2011 Mar 14;6(3):e17800. doi: 10.1371/journal.pone.0017800 (PMC3056789; doi:10.1371/journal.pone.0017800)

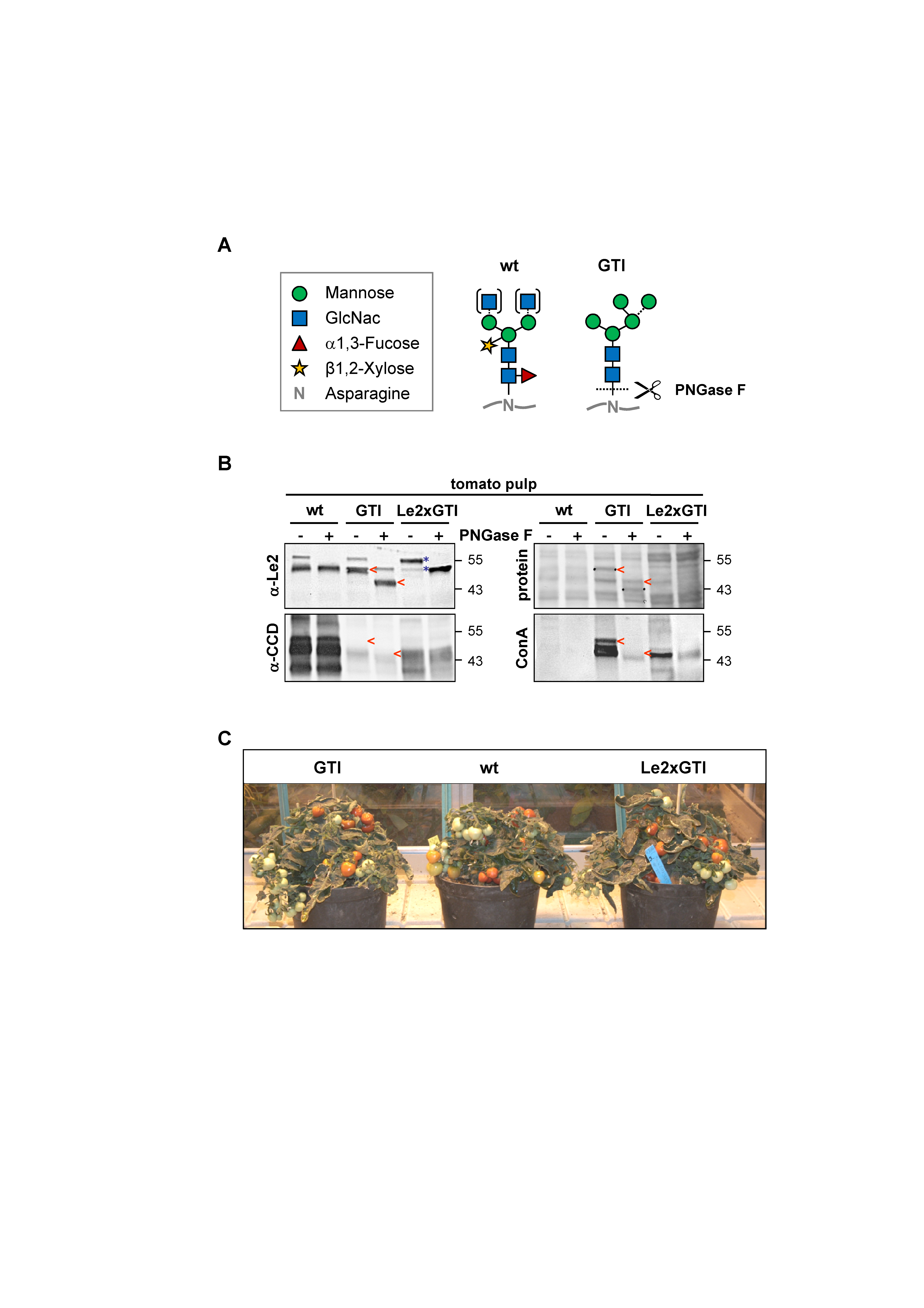

Supplement: Figure S1 — PNGase-F treatment of tomato fruit extracts verifies efficient Lyc e 2-silencing. (TIFF) [file pone.0017800.s002.tiff]

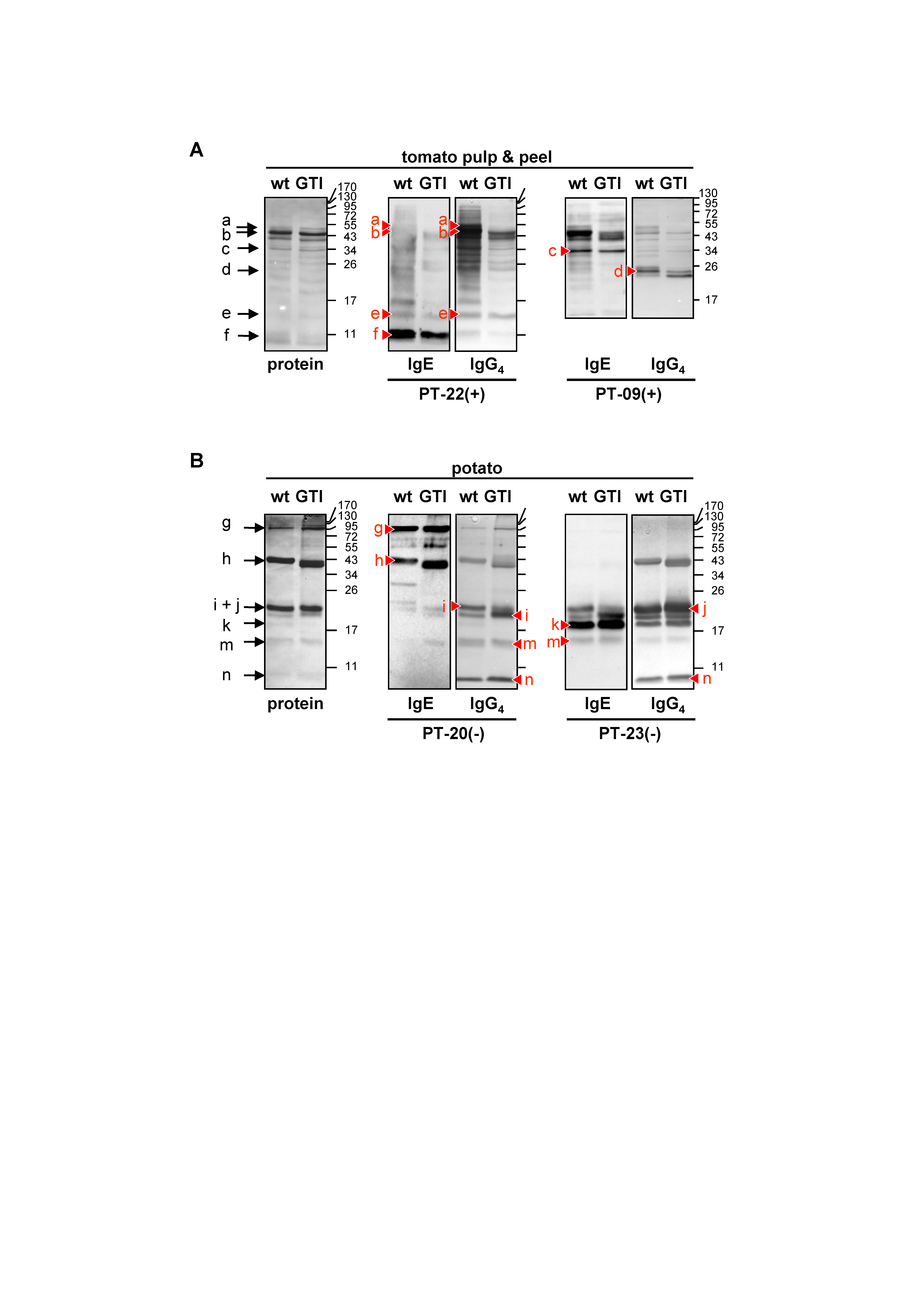

Supplement: Figure S2 — GNTI-silenced plants maintain CCD-independent specific IgE and IgG4 binding. (TIFF) [file pone.0017800.s003.tiff]

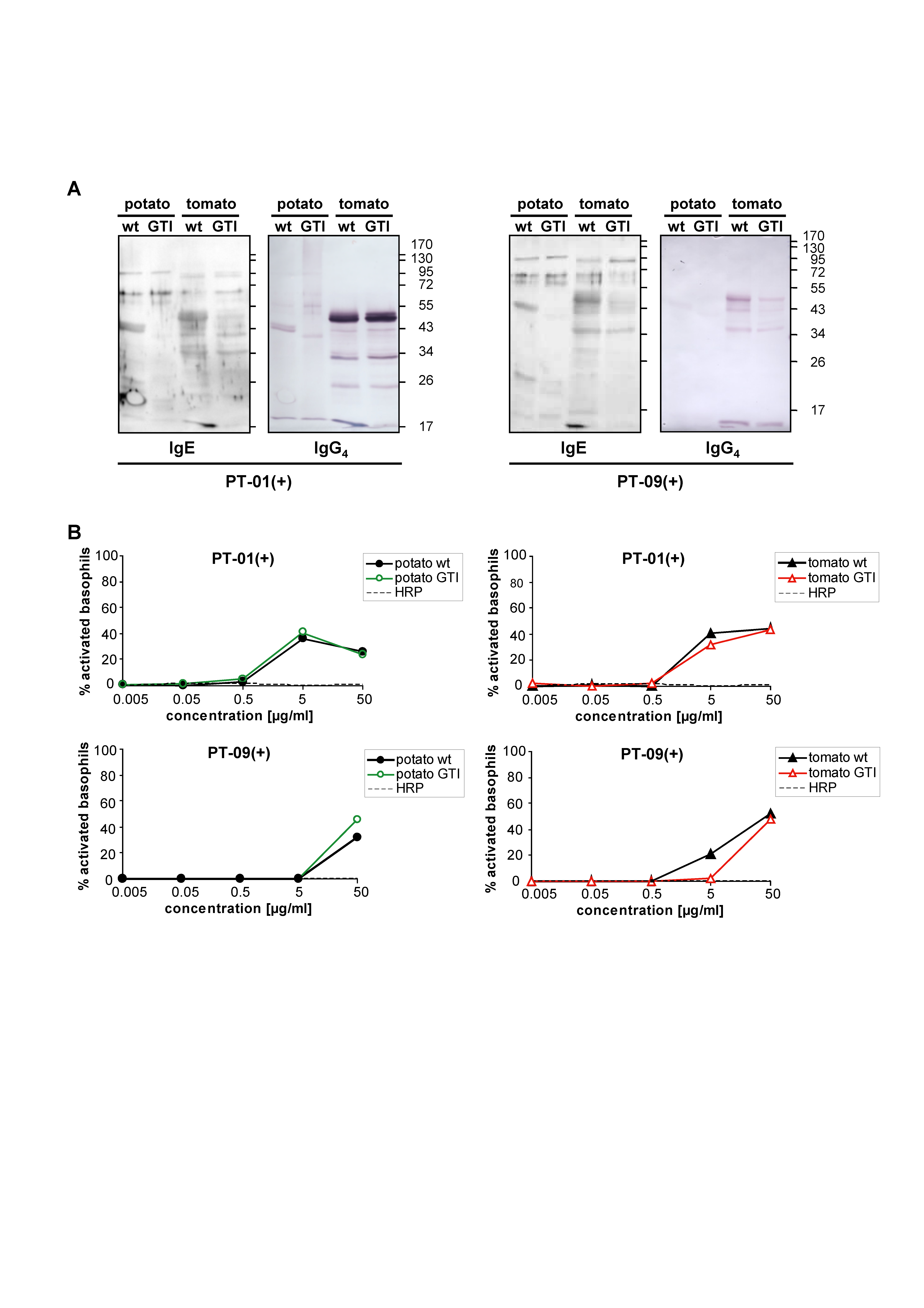

Supplement: Figure S3 — Basophil activation in borderline CCD-positive potato/tomato-allergic patients. (TIFF) [file pone.0017800.s004.tiff]
